# Do gifts increase consent to home-based HIV testing? A difference-in-differences study in rural KwaZulu-Natal, South Africa

**DOI:** 10.1093/ije/dyw122

**Published:** 2016-12-03

**Authors:** Mark E McGovern, Kobus Herbst, Frank Tanser, Tinofa Mutevedzi, David Canning, Dickman Gareta, Deenan Pillay, Till Bärnighausen

**Affiliations:** 1 CHaRMS – Centre for Health Research at the Management School, Queen's University Belfast, Northern Ireland; 2 UKCRC Centre of Excellence for Public Health (Northern Ireland); 3 Africa Health Research Institute, Mtubatuba, South Africa; 4 School of Nursing and Public Health, University of KwaZulu-Natal, Durban, South Africa; 5 Centre for the AIDS Programme of Research in South Africa, CAPRISA, University of KwaZulu-Natal, Congella, South Africa; 6 Department of Global Health and Population, Harvard TH Chan School of Public Health, Boston 02115, MA, USA; 7 Harvard Center for Population and Development Studies, Cambridge 02144, MA, USA; 8 Institute of Public Health, University of Heidelberg, Heidelberg, Germany

**Keywords:** home-based HIV testing, gift-voucher intervention, difference-in-differences (DD) analysis, rural South Africa

## Abstract

**Background:** Despite the importance of HIV testing for controlling the HIV epidemic, testing rates remain low. Efforts to scale up testing coverage and frequency in hard-to-reach and at-risk populations commonly focus on home-based HIV testing. This study evaluates the effect of a gift (a US$5 food voucher for families) on consent rates for home-based HIV testing.

**Methods:** We use data on 18 478 individuals (6 418 men and 12 060 women) who were successfully contacted to participate in the 2009 and 2010 population-based HIV surveillance carried out by the Wellcome Trust's Africa Health Research Institute in rural KwaZulu-Natal, South Africa. Of 18 478 potential participants contacted in both years, 35% (6 518) consented to test in 2009, and 41% (7 533) consented to test in 2010. Our quasi-experimental difference-in-differences approach controls for unobserved confounding in estimating the causal effect of the intervention on HIV-testing consent rates.

**Results:** Allocation of the gift to a family in 2010 increased the probability of family members consenting to test in the same year by 25 percentage points [95% confidence interval (CI) 21–30 percentage points; *P* < 0.001]. The intervention effect persisted, slightly attenuated, in the year following the intervention (2011).

**Conclusions:** In HIV hyperendemic settings, a gift can be highly effective at increasing consent rates for home-based HIV testing. Given the importance of HIV testing for treatment uptake and individual health, as well as for HIV treatment-as-prevention strategies and for monitoring the population impact of the HIV response, gifts should be considered as a supportive intervention for HIV-testing initiatives where consent rates have been low.

## Introduction

High levels of participation in HIV testing are important for clinical and public health disease management, and also for policy—to gain information about the spread of the HIV epidemic and to evaluate the population effectiveness of HIV interventions.^1^ However, despite the accepted importance of HIV testing, testing rates remain low in many countries.^2^ HIV treatment-as-prevention will require an increase in the frequency and coverage of testing in order to place individuals on treatment rapidly after diagnosis.^3^ Those who test as HIV-negative can be counselled and referred to prevention services to reduce the risk of acquiring HIV. New waves of HIV testing will need to reach those populations who have not previously tested,^4^ particularly if the UNAIDS goal of 90% of people living with HIV knowing their status by 2020 is to be met.^5^

Home-based testing is a promising approach for supporting the goal of raising HIV-testing rates,^6^^,^^7^ and can be used to target populations that are otherwise hard to reach,^8^ in particular those who have never tested for HIV in health care facilities. The WHO has recently endorsed home-based HIV testing as an important major approach for ‘overcoming some of the barriers of access to testing services and providing testing to individuals who might not otherwise seek services’.^9^ However, rates of participation in HIV testing during home visits vary widely across communities.^6^

There is some evidence that incentives can be effective in increasing HIV-testing rates in clinical settings or voluntary counselling and testing centres.^10^^,^^11^ Cash transfers have been attracting growing attention for improving health outcomes,^12^ but, as we outline in Appendix 1, gifts have a number of advantages over conditional cash transfers, including that they may be less intrusive on decision making, less likely to replace intrinsic with extrinsic motivation (potentially jeopardizing participation when the incentive is removed), and less likely to compromise informed consent because of undue inducement.

Several surveys with a home-based HIV-testing component have included gifts as part of the routine survey procedure,^7^^,^^13^^,^^14^ e.g. to compensate participants for their time spent answering survey questions. Such gifts have included bars of soap,^7^ money,^13^ and bednets and water purification.^14^ However, to our knowledge, ours is the first study evaluating the effect of a gift on home-based HIV-testing consent.

The gift intervention we evaluate in this paper takes place in the context of home-based HIV testing in an annual population-based HIV surveillance, which has been running for over a decade and where the HIV survey fieldworkers are members of the local community.^15^ Thus, the intervention takes place in the context of ongoing interaction between the community and the HIV survey team. We hypothesize a positive effect of the gift voucher in this setting.

## Methods

### Intervention

The Africa Health Research Institute (AHRI) is one of the five major overseas programmes funded by the UK medical research charity, the Wellcome Trust. AHRI has carried out population-based health and demographic surveillance of an entire rural, Zulu-speaking community in KwaZulu-Natal, South Africa, for over a decade. Since 2003, this surveillance has included an annual population-based HIV surveillance; since 2007, all residents aged 15 and over have been eligible for participation in the surveillance. The HIV data, collected through home-based testing, have been widely used to describe the evolution of the epidemic and its impact.^15–17^ HIV prevalence is high in this community (24% in 2010), and rates of participation in HIV testing are low (41% in 2010),^18^ making this hyperendemic setting one of the most policy-relevant worldwide for evaluating the effectiveness of interventions to raise participation in HIV testing.

**Figure 1.**
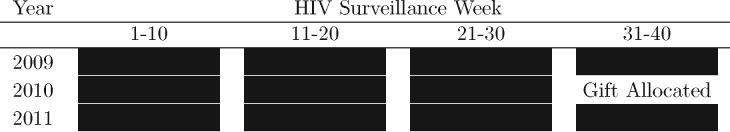
Summary of gift intervention timing. HIV surveillance took place as normal during 2009 and 2011, and the first 30 weeks of the 2010 surveillance. The voucher was allocated in the final 10 weeks of the 2010 surveillance only, and not in 2009 or 2011.

With the goal of increasing HIV-testing consent in the community, and in consultation with the local Community Advisory Board, a gift intervention was implemented in the 2010 HIV surveillance. A food voucher worth 50 South African Rand (approximately US$5 at the time) was given to all families whose members were contacted for testing in the final 10 weeks of the surveillance. One voucher was given per family and voucher receipt was not conditional on family members consenting to test. The presentation of the gift was made at first contact with the family, so all members of that family were considered to have received the intervention. The gift was given to the head of the family or, if he or she was not present, to the next person defined by hierarchical ranking of family members. The total cost of all vouchers allocated was 68 000 South African Rand. Further details of the gift intervention and the data are provided in Appendix 1. Figure 1 gives an outline of how the intervention was implemented.

### Difference-in-differences estimation of causal effects

Because the gift was not randomly assigned, we adopted a quasi-experimental design—difference-in-differences (DD)^19^^,^^20^—in order to test the hypothesis that the intervention increased participation in HIV testing. The DD approach is to evaluate the *difference* between the change in the consent rate over time for the intervention group (i.e. before and after the receipt of the gift) and the change in the consent rate over the same period of time in the control group (who did not receive the gift). Because we focus on changes within these groups over time, we are able to account for all time-invariant characteristics of the intervention group. Assuming that the trend in consent rates among those who received the voucher would have been the same, in the absence of the treatment, as the trend in consent rates among those who did not receive the voucher, we can estimate the causal effect of the intervention. Because the gift was allocated for operational reasons, and not based on the characteristics of the individual participants or families, we believe this ‘parallel trend’ assumption is reasonable in our application.

In this analysis, the treatment group consists of all members of families that received the gift voucher, whereas the control group consists of all members of families that did not receive the voucher. We define a family to have received the voucher if any family member was contacted by the surveillance team and presented with the gift.

The intervention was implemented so that only families contacted in the final 10 weeks of the surveillance received the gift. Because at least one member of almost all families was successfully contacted in the AHRI surveillance in each surveillance round, and because the gift was presented at the first contact with an available family member and handed over directly through personal contact, there is no reason why all families could not have received the gift and thus be in the treatment group.

We implement the DD approach using a linear probability model which allows us to estimate the difference in the change in consent rates for treatment and control groups while adjusting for the characteristics of respondents recorded as part of the surveillance, and to calculate confidence intervals in a straightforward manner. The individual is the analytic unit of interest, and we have two time periods and thus two observations for each individual (2009 and 2010). Using the pooled individual-level datasets for the 2009 and 2010 HIV surveillance surveys, our outcome of interest is a binary indicator which describes whether the individual consented to an HIV test, *Consent_ij_* for person *i* in year *j.* The regression specification for our main analysis is as follows (equation 1):

(1) $$Consent_{ij}=\beta_{1}{\left(Year=2010\right)}_{j}+\beta_{2}{\left(Intervention=1\right)}_{i}+\beta_{3}\left(Year=2010\right)*{\left(Intervention=1\right)}_{ij}+X_{ij}\theta +\mu_{ij}.$$

The DD estimate of effect of the voucher is an interaction between a binary variable that takes the value 1 if individual *i* was a member of a family that received the gift (*Intervention* = 1) and a binary indicator for whether the outcome was measured in 2010 (*Year* = 2010). *µ_ij_* is an individual-level error term. Because the voucher was allocated at the family level, we adjust our standard errors for correlation between family members. We stratify our models by sex to allow for differential effects in men and women.

As we include a separate indicator variable for calendar year of interview, the coefficient *β*_3_ captures the additional change in consent rates associated with being in the intervention group, once the trend in the control group has been accounted for. We adjust for the observed time-varying socio-demographic characteristics of respondents in *X_ij_.* These characteristics include age group, marital status, mother is alive, father is alive, education, type of location, month of interview, electricity in the household, household fuel, household assets index, running water in the household, and flush toilet in the household.

In order to account for potential spatial clustering, we adjust for *isiGodi* of residence (an *isiGodi* is a demarcated traditional ward within the area of jurisdiction of a traditional council in the traditional Zulu leadership structure) using indicator variables, as well as distance from the household to the nearest clinic, secondary school, primary school, level-1 road and level-2 road. Because our data are longitudinal and we observe whether the respondents consent to test in both years, we can additionally match respondents in 2010 to themselves in 2009 by including an individual-level fixed effect (indicator variable) as a covariate. Matching individuals to themselves has the benefit of accounting for all individual-level time-invariant observed and unobserved confounders.

The causal inferences literature typically uses least squares linear regression for binary outcomes with longitudinal data because this allows estimates of the risk difference to be obtained directly.^20^ The linear probability model has the added advantage that, unlike logistic models, it does not drop individuals who do not exhibit a change in the outcome over time in longitudinal fixed-effects estimation.^21^ We do, however, also estimate odds ratios from logistic models, and find very similar results (which are described in detail in Appendix 1).

### Sample size

**Figure 2.**
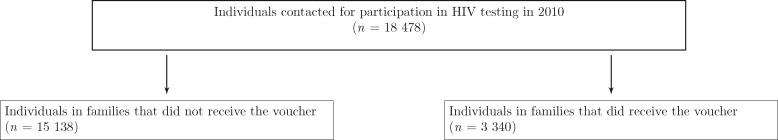
Summary of analysis sample. 18 478 individuals were contacted for an HIV test in the AHRI HIV Surveillance cohort in 2009 and 2010. 3 340 individuals were members of a family that received the voucher in 2010. The voucher was allocated in 2010 only, and not in 2009 or 2011.

In total, 18 478 eligible residents (12 060 women and 6 148 men) in the AHRI HIV Surveillance were contacted to undertake a home-based HIV test in 2009 and 2010. All of these residents are included in the main analysis. In total, 3 340 individuals were members of a family that received the voucher in 2010. Figure 2 shows the sample selection for this main analysis. In a subsequent additional analysis, we also include those who were contacted for consent in 2009, 2010 and 2011 (*n* = 13 488). Table A1 in Appendix 1 shows descriptive statistics for the other variables we use as covariates in the analysis.

## Results

### Unadjusted results for HIV-testing consent

Unadjusted estimates of rates of consent to participate in HIV testing in 2009 and 2010 are shown in Table 1

**Table 1.** Testing participation rates by control and intervention groups in 2009 and 2010

		2009				2010		
		Number declined to test	Number consented to test	% Consented to test (95% CI)		Number declined to test	Number consented to test	% Consented to test (95% CI)
**Men**								
Intervention		907	299	25% (22–27%)		708	498	41% (39–44%)
Control		3 560	1 652	32% (30–33%)		3 643	1 569	30% (29–31%)
**Women**								
Intervention		1 411	723	34% (32–36%)		899	1 235	58% (56–60%)
Control		6 082	3 844	39% (38–40%)		5 695	4 231	43% (42–44%)

The percentage consenting to test for HIV in 2009 and 2010 for the control group (members of families that did not receive the food gift voucher in 2010) and the intervention group (members of families that did receive the food gift voucher in 2010) is shown, along with corresponding 95% confidence intervals, which are rounded to the nearest percent. All families in the last 10 weeks of the 40-week surveillance were allocated to receive an unconditional food gift voucher worth US$5 at the first contact with the family. The voucher was allocated in 2010 only. Of the total 6 418 men in the analysis sample, 1 206 (19%) were in the intervention group. Of the total 12 060 women in the analysis sample, 2 134 (18%) were in the intervention group. CI = confidence interval.

. Men and women in the control group who did not receive the intervention in 2010 both show relatively little difference in consent rates in 2010 compared with 2009. The consent rate for men in the control group in 2009 was 32% [95% confidence interval (CI) 31–33%], compared with 30% in 2010 (95% CI 29–31%). For women, the consent rate in the control group in 2009 was 39% (95% CI 38–40%), compared with 43% in 2010 (95% CI 42–44%). In contrast, consent rates among the intervention group increased substantially. For men, the consent rate for the intervention group in 2009 was 25% (95% CI 22–27%), compared with 41% (95% CI 39–44%) in 2010. For women, the consent rate in the intervention group in 2009 was 34% (95% CI 32–36%), compared with 58% in 2010 (95% CI 56–60%). Note that the consent rate in 2009 was substantially lower for the intervention than the treatment group. This finding supports our analytic strategy of adjusting for baseline data, because intervention and control groups had different consent rates at the beginning of the study period.

### DD estimation results

The main results from the DD analysis are presented in Table 2

**Table 2.** Regression results for the effect of receiving the gift voucher on consent to participate in testing (difference-in-differences)

Outcome	Consent to HIV test			
Model	Linear regression DD	Linear regression DD	Linear regression DD	Linear regression DD
Covariates:	No covariates	Baseline (2009) observed characteristics only	All observed characteristics	All observed characteristics
				+ individual fixed effects
	Gift-intervention adjusted risk difference in percentage points (95% CI)			
Sample: Women and men combined (*n* = 18 478)	19 (17–22)*	19 (17–22)*	19 (16–23)*	25 (21–30)*
Sample: Women (*n* = 12 060)	20 (17–23)*	20 (17–23)*	20 (16–24)*	25 (20–30)*
Sample: Men (*n* = 6 418)	18 (14–21)*	18 (14–22)*	18 (13–23)*	27 (20–33)*

* Significant at *P* < 0.01. All families in the last 10 weeks of the 40-week surveillance were allocated to receive an unconditional food gift voucher worth US$5 at the first contact with the family. The voucher was allocated in 2010 only. Consent data for individuals who were contacted to take an HIV test in 2009 and 2010 were pooled and the effectiveness of the voucher is the difference-in-differences (DD) estimate associated with being in the intervention group in the year 2010. Each column shows the adjusted risk difference for consenting to test in percentage points associated with being a member of a family which received the gift voucher. 95% CIs (rounded to the nearest percent) are shown in parentheses. The first column shows the DD estimates without adjusting for observed individual-level characteristics. The estimates in the second column are adjusted for baseline 2009 characteristics only, whereas the estimates in the third column are adjusted for observed characteristics in 2009 and 2010. The estimates in the final column are additionally adjusted for an individual-level fixed effect so that respondents in 2010 are matched with themselves in 2009. All models are linear regressions and CIs are adjusted for clustering at the family level. Descriptive statistics for covariates are listed in appendix Table A1, and include: age group, marital status, mother is alive, father is alive, education, location (*isiGodi*), type of location, electricity in the household, household fuel, household-asset index, running water in the household, flush toilet in the household and distance to nearest: clinic, secondary school, primary school, level 1 road, level 2 road. The full regression table for the model in column 4 showing these coefficients is presented in the appendix Table A2. Of the total 18 478 individuals in the analysis sample, 3 340 (18%) were in the intervention group. Of the total 6 418 men in the analysis sample, 1 206 (19%) were in the intervention group. Of the total 12 060 women in the analysis sample, 2 134 (18%) were in the intervention group. CI = confidence interval.

. We consider a number of alternative model specifications in order to test the robustness of our results. In column 1 of Table 2, we do not adjust for any observed characteristics of respondents whereas, in column 2, we adjust for baseline characteristics only. Column 3 adjusts for observed characteristics in both years, whereas column 4 additionally adjusts for individual-level fixed effects. Overall, estimates are similar regardless of the specification. The final column is our preferred model because it adjusts for time-invariant unobserved confounding at the individual level. For the combined sample of men and women, these estimates indicate that being a member of a household that received the voucher increased the probability of the individual consenting to an HIV test by 25 percentage points (95% CI 21–30 percentage points; *P* < 0.001). Estimates are also very similar when we stratify by sex. Among men, the point estimate for the gift-voucher effect is 27 percentage points (95% CI 20–33 percentage points; *P* < 0.001). Among women, the point estimate is 25 percentage points (95% CI 20–30 percentage points; *P* < 0.001). We show the full set of results for the model in the final column of Table 2; we show the coefficient estimates for all other covariates in the appendix.

### Secondary analysis for HIV-testing consent in 2011

**Figure 3.**
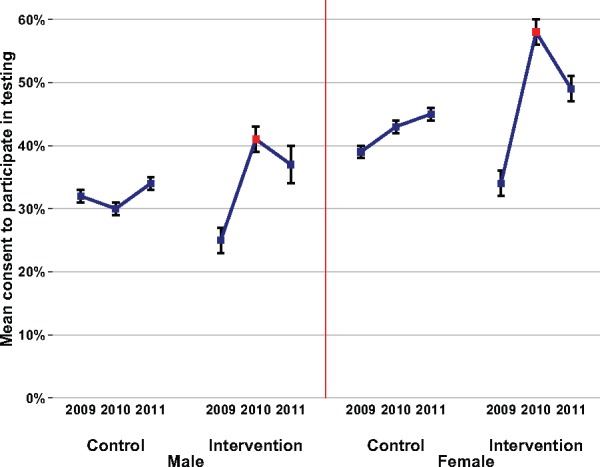
Rates of consent to participate in testing 2009–2011 by intervention group. Rates of consent to test for HIV in 2009, 2010 and 2011 are shown. For the 2009 and 2010 estimates, the sample is restricted to those contacted for consent to provide blood for an HIV test in 2010 and 2009 (*n* = 18 478). For the 2011 estimates, the sample is restricted to those contacted for consent to provide blood for an HIV test in 2011 and 2010 and 2009 (*n* = 13 488). All families in the last 10 weeks of the 40-week surveillance in 2010 were allocated to receive an unconditional food gift voucher worth US$5 at the first contact with the family. The voucher was allocated in 2010 only, and not in 2009 or 2011. The intervention group is defined as being a member of a family that received the food voucher in 2010, whereas the control group is defined as being a member of all other families who did not receive the voucher in 2010. 95% confidence intervals (CIs) are also shown, rounded to the nearest percent. Of the total 18 478 individuals in the analysis sample in 2010, 3 340 (18%) were in the intervention group. Of the total 6 418 men in the analysis sample in 2010, 1 206 (19%) were in the intervention group. Of the total 12 060 women in the analysis sample in 2010, 2 134 (18%) were in the intervention group.

As a secondary analysis, we determine whether HIV-testing participation rates continued to be affected after the gift intervention was removed in 2011. These results are shown in Figure 3. Our estimates for consent rates in 2011 are based on those who were contacted for HIV testing in all three years (2009, 2010 and 2011). The sample for this secondary analysis (*n* = 13 488) is smaller than the sample for the main analysis because not all 2010 residents were contacted in 2011. In this surveillance, the vast majority of eligible residents are successfully contacted, and testing HIV-positive in a previous wave is not associated with not being contacted in a subsequent year.^18^ All residents in the surveillance area aged 15 years and older are eligible for HIV testing in each round of the longitudinal surveillance, independent of HIV status and previous testing history. However, any respondents who had out-migrated in 2011 would not have been eligible for participation in that year. We do not know whether this subgroup of out-migrants would have consented to test if they had been a resident in 2011, and whether consent rates for this subgroup would differentially depend on having received the gift.

For men, the consent rate for the intervention group in 2011 was 37% (95% CI 34–41%), which is slightly lower than in 2010, but substantially higher than in 2009. Likewise, for women, the consent rate for the intervention group in 2011 (49%, 95% CI 46–51%) was slightly lower than in 2010, but substantially higher than in 2009.

### Consequences for HIV prevalence estimates

An additional benefit of interventions that increase consent rates for HIV testing is that information on the intervention effect can be used to evaluate the extent of selection bias in the sample. This analytical opportunity arises because the intervention, if successful, will increase consent rates by persuading individuals to test for HIV who would ordinarily have refused to test. Thus, the gift intervention can be used to estimate HIV prevalence for the subgroup of residents for whom, without the intervention, HIV status would likely not have been observed in the year of the intervention (2010). In Table 3

**Table 3.** HIV prevalence estimates in the AHRI HIV Surveillance in 2009 and 2010

		2009				2010		
		Number HIV-negative	Number HIV-positive	% HIV-positive (95% CI)		Number HIV-negative	Number HIV-positive	% HIV-positive (95% CI)
**Men**								
Intervention		230	68	23 (18–28%)		357	140	28 (24–32%)
Control		1 448	203	12 (11–14%)		1 339	210	14 (12–15%)
**Women**								
Intervention		477	243	34 (30–37%)		717	509	42 (39–44%)
Control		3071	767	20 (19–21%)		3 263	927	22 (21–23%)

All families in the last 10 weeks of the 40-week surveillance were allocated to receive an unconditional food gift voucher worth US$5 at the first contact with the family. The voucher was allocated in 2010 only. The intervention group is defined as being a member of a family that received the food voucher in 2010, whereas the control group is defined as being a member of all other families who did not receive the voucher in 2010. Mean HIV prevalence rates in the control and intervention groups in 2009 and 2010 are shown, stratified by sex. 95% confidence intervals (CIs) are also shown, rounded to the nearest percent. Of the total 18 478 individuals in the analysis sample, 3 340 (18%) were in the intervention group. Of the total 6 418 men in the analysis sample, 1 206 (19%) were in the intervention group. Of the total 12 060 women in the analysis sample, 2 134 (18%) were in the intervention group. 30% (95% CI 29–31%) of men in the control group consented to test in 2010, compared with 41% (95% CI 39–44%) in the intervention group. Among women in 2010, 43% in the control group consented to test (95% CI 42–44%) compared with 58% in the intervention group (95% CI 56–60%). In 2009, 32% (95% CI 31–33%) of men in the control group consented to test, compared with (25% (22–27%) in the intervention group. Among women in 2009, 39% in the control group consented to test (95% CI 38–40%), compared with 34% in the intervention group (95% CI 32–36%) AHRI = Africa Health Research Institute.

, we compare HIV prevalence among members of families that received the intervention in 2010 to members of the same families in 2009, before they received the intervention.

For men, HIV prevalence among the intervention group in 2010 was 14% (95% CI 12–15%), which did not increase much relative to the control group in 2009, which had an HIV prevalence of 12% (95% CI 11–14%). In contrast, HIV prevalence among the intervention group for men increased to 28% (95% CI 24–32%) in 2010, from 23% (95% CI 18–28%) in 2009. Similarly, there was a much larger increase in HIV prevalence observed for women in the intervention group, compared with the control group. HIV prevalence among women in the control group was 22% (95% CI 21–23%) in 2010, compared with 20% (95% CI 19–21%) in 2009. In contrast, HIV prevalence among women in the intervention group was 42% (95% CI 39–44%) in 2010, compared with 34% (95% CI 30–37%) in 2009. These results indicate that the group of people who are motivated by the gift voucher to participate in HIV testing, but ordinarily would not have tested, are more likely to be HIV-infected than those who consent to testing without the gift intervention.

## Discussion

Following the recent target set by UNAIDS of 90% of HIV-positive individuals knowing their status by 2020, identifying effective interventions for increasing and maintaining participation in HIV testing will become an increasingly important topic. In an HIV hyperendemic community in South Africa, we find that a gift voucher given to families as part of home-based HIV testing in a population-based HIV surveillance increased consent to participate in HIV testing by 25 percentage points.

An important question is whether interventions that transfer items of monetary value crowd out intrinsic motivation, potentially resulting in participation rates that are lower than baseline after the incentive is removed.^22^^,^^23^ Our results show no evidence of crowding out. In contrast, we find a substantial positive effect in the year following the implementation of the intervention, i.e., when the gift voucher was no longer given. This finding conforms with theories of behaviour that predict that conditional cash transfers replace intrinsic with extrinsic motivation but that gifts – which are by definition unconditional – do not lead to such a change in the pattern of motivation. This finding also indicates that focusing analysis solely on the year in which the intervention took place can lead to an underestimate of the full gift-voucher effect.

The effect of the gift compares favourably to other interventions designed to increase consent to HIV testing. According to a recent review of strategies to increase testing, eight studies reported an (unadjusted) risk difference for men of 25 percentage points or less; five were in the 26–35 percentage-point range; and five reported a risk difference of 36 percentage points or more.^24^ Of seven studies involving incentives specifically for HIV/STI testing,^25^ only one had a larger risk difference (at 43 percentage points) than the one we found for this gift intervention.^26^

The value of the gift voucher was relatively small, and our results are therefore consistent with findings that ‘micro-gifts’ can substantially improve health care-seeking behaviours.^27^ Our results are also consistent with the literature that emphasizes that, by providing an immediate reward or payoff, even seemingly low-value interventions can be successful at altering behaviour that generally only has a long-run benefit to the person making the behaviour change. In addition, the effectiveness of the gift for home-based HIV testing, even though it was unconditional, highlights the role of gifts in signalling social norms for reciprocity.^28^ In a longitudinal setting with repeated visits, mutual trust between participants and the survey team is likely to be important.^29^

Given the importance of increasing the frequency and coverage of HIV testing to make further progress in reducing HIV-related mortality in hyperendemic communities, and to support potential future HIV treatment-as-prevention programmes, the problem of refusal rates in household and surveillance surveys is likely to become increasingly important and relevant for health policy-makers charged with implementing public health HIV-intervention programmes. HIV-related mortality remains high in this community, and it largely occurs among individuals who have never accessed the local HIV-treatment programme.^30^ Expanding the coverage of HIV testing can likely contribute to ensuring that all sub-populations benefit from HIV treatment. Expanding testing is also likely to have complex effects on behaviour.^31^^,^^32^ Future research examining the potential for gift interventions to increase linkage to care would be beneficial for identifying pathways to UNAIDS testing and treatment targets.^5^^,^^33^^,^^34^

We have illustrated how a micro-gift can be used to evaluate the presence of selection bias in the data by estimating HIV prevalence in the sample of people who would ordinarily refuse to test for HIV but who are motivated by the gift to participate in testing. Formal modelling using Heckman-type selection models is required to evaluate this finding further and provide estimates of population HIV prevalence that adjust for selection bias.^35–37^

The DD design has important advantages over regression or matching-based methods typically used to analyse observational data. By comparing the change in outcomes of an intervention group (which received the treatment of interest) and a control group (which did not), we are able to account for group-level unobserved confounders that are time-invariant. With quasi-random variation in an exposure due to, for example, a policy change or intervention, quasi-experimental methods such as DD provide plausible opportunities to examine questions of causal inference. Quasi-experimental approaches are also valuable because they allow us to estimate causal effects in situations in which randomized control trials (RCTs) are not feasible, such as for many health policy changes implemented by government, or when RCTs are not practical for ethical reasons. The literature in epidemiology which has adopted quasi-experimental approaches, such as DD, is currently growing, and there is great potential for implementing this methodology increasingly widely for causal inference in public and global health research.^38^^,^^39^ One reason for this potential is the current expansion in the availability of large-scale population-based surveys and administrative data.^40^

### Limitations

As the monetary amount of the micro-gift was fixed, we cannot evaluate whether the gift effect would have been similar even if the value had been lower. Alternatively, a more valuable gift could have been even more effective. Likewise, we cannot evaluate whether non-monetary gifts are more effective than monetary gifts. Several other surveys have included gifts as part of the routine survey procedure.^7^^,^^13^^,^^14^ Comparison of unconditional gifts with conditional incentives, and comparison of gifts and incentives of different values and types in other settings will further improve our evidence for designing optimal interventions to improve the uptake of HIV testing.

Although the DD approach cannot rule out confounding due to unobserved factors which change over time, we do control for a wide range of potential time-varying confounders, such as age, marital status and household-asset index. We do not observe the HIV status of individuals who refuse to test in both 2009 and 2010, and this is plausibly related to consent,^35^^,^^41^^,^^42^ but we would only expect a small proportion of people to change their HIV status from one year to the other, as the overall incidence rate in this population is about 3 per 100 person-years.^43^

The intervention was conducted in the context of an ongoing annual health and demographic surveillance within a hyperendemic community. It is possible that an alternative form of gift (such as a conditional cash transfer) could be more effective in another context, such as in a single survey. In addition, individual consent rates in the AHRI HIV Surveillance prior to the gift intervention were relatively low compared with some other surveys in South Africa,^44–46^ and consequently there was substantial scope for raising testing rates in this study community. For these reasons, it would be interesting to examine whether the results found here extend to other settings.

## Conclusions

Gifts can be highly effective in increasing consent to HIV testing in hyperendemic communities in South Africa. HIV testing is important for treatment uptake and individual health, as well as for HIV treatment-as-prevention strategies and for monitoring the population impact of the HIV response. Gifts should thus be considered as a supportive intervention for HIV-testing initiatives where consent rates are low.

## Supplementary Material

Supplementary Data
