# Supplementary material for: Do gifts increase consent to home-based HIV testing? A difference-in-differences study in rural KwaZulu-Natal, South Africa
Source: Int J Epidemiol. 2016 Dec 3;45(6):2100–9. doi: 10.1093/ije/dyw122 (PMC5841834; doi:10.1093/ije/dyw122)
Supplement: Supplementary Data [file dyw122_supplementary_data.zip › ije-2015-06-0745-File002.pdf]

Do gifts increase consent to home-based HIV testing? Evidence from rural KwaZulu-Natal, South Africa

Supplementary Material

Advantages of Gifts over Conditional Cash Transfers

Many methods to increase consent to HIV testing have been proposed and tested,[1–5] including financial incentives, or conditional cash transfers.[6–8] An alternative to conditional cash transfers in altering this balance are gifts, which have a number of potentially desirable characteristics.[9,10] First, gifts can be considered as less intrusive on participants' decision making process than conditional cash transfers because they do not mandate a particular course of action which is assumed to be in their best interest, but instead trusts people to make the optimal choices for themselves.[11] A particular concern about conditional cash transfers is that they may replace intrinsic motivation with extrinsic motivation, potentially jeopardizing participation in the activity or behaviour in the future when the incentive is removed.[12,13] Second, conditional cash transfers may be seen as being an undue inducement if the amount offered is too high. Particularly in low resource settings, the process of informed consent will be compromised if the incentive to participate does not realistically offer the option to decline. In addition, incentives can be unethical if they lead to poor judgement, or the assumption of greater risks than would be the case in the absence of the inducement.[14] Third, conditional cash transfers can imply a hierarchal relationship between the implementers of an intervention and potential intervention participants. In contrast, gifts reflect more reciprocal relationships.[15,16] Reciprocity is likely to be an important mechanism for increasing consent rates in home-based testing. Even though the gift is formally unconditional, a general expectation is often formed of a return being due to the gift-giver, which may be reinforced by the social norms within a community.[17–20] Presenting an offer for

HIV testing provides the gift recipient with the immediate opportunity to fulfil this expectation. Finally, gifts are likely to be easier to implement and have lower transaction costs, because unlike conditional cash transfers, they do not require monitoring of outcomes.[11]

Data description

Data in this paper are from the longitudinal population-based surveillance system carried out by the Wellcome Trust Africa Centre for Health and Population Studies, which has carried out home-based HIV testing among residents of a predominantly rural community in KwaZulu-Natal, South Africa, since 2003. The surveillance area covers roughly 434 km^2 and includes both an urban township and peri-urban settlements. Overall, this community is one of the poorest in all of South Africa.[21] Twice per year, information is collected from a key family informant on the socio-demographic characteristics of respondents and households, including births, deaths and migration. These data provide information on the physical structures, household characteristics and events, and individual members and their relationships. The annual Africa Centre HIV surveillance cohort is nested within this household survey, and since 2007 all residents over the age of 15 are eligible for HIV testing. Potential HIV survey participants are visited annually by teams of two trained fieldworkers. Written consent is sought, and following this a blood sample is collected by finger prick, and the dried blood spots are prepared in accordance with UNAIDS and WHO guidelines for HIV testing.[21] The population-based survey also involves continuous collection of data from families and eligible participants. If an individual eligible for HIV testing is not present during the home visits by the survey team, the team will make follow-up visits to contact this individual at a later date (up to three subsequent visits).

Further Details of the Gift Intervention

With the goal of raising HIV testing consent in the community, Africa Centre surveillance researchers presented the idea of using a conditional cash transfer as an intervention to the Africa Centre Community Advisory Board. The Community Advisory Board instead advocated for the use of a gift because gift-giving is appropriate and common among traditional African societies,[20] and because the conditional cash transfer was perceived to be intrusive on people's decision making. The gift intervention was then implemented in the form of a voucher worth 50 South African Rand (approximately GBP3, US$ 5), which could be used to purchase food items at the local store of a large South African grocery chain. Families in which individual members were contacted for consent to participate in HIV testing in the final 10 weeks of the 2010 HIV surveillance (out of the 40 week-long survey conducted during the year as a whole) were provided with the gift. The gift was presented as a "thank you" to the community members for their continued participation in the Africa Centre surveillance activities following the ten-year anniversary of the Africa Centre, and was given at the beginning of the interview, and therefore was not conditional on consent to HIV testing. On first contact with the family, the gift was presented to the household head, or if not available, the next highest ranking person on a hierarchical list of residents. Overall, 47% of vouchers went to the family head, 21% to their spouse or partner, 15% to their child, and the remaining 17% to other family members. About one third (38%) of the population eligible for HIV testing lived in a family which received the gift intervention.

This approach of using a micro-gift was specifically recommended by the Africa Centre Community Advisory Board as a more culturally appropriate alternative to a conditional cash transfer. Indeed, the use of a micro-gift is likely preferable in this context because it signals a reciprocal relationship and not a hierarchical one implied by a conditional monetary transaction. In contrast, a conditional transaction could potentially induce psychological

feelings of lack of control and anxiety with subsequent adaptive behaviour. Anecdotal feedback obtained from the surveillance fieldworkers was that they felt the voucher facilitated their role because it enabled them to go to the field with the opportunity to offer something concrete to participants for the first time, rather than in previous rounds of the surveillance where they were exclusively requesting something from participants (their information).

Statistical approach – difference-in-differences

The voucher was allocated according to week of interview, which is correlated with location. Although there is enough variation within districts (Isigodi) to allow us to control for this level of aggregation in the analysis, one potential source of bias is that there may be unobserved confounders at a smaller geographic scale, for example due to the geographic clustering of HIV within communities.[26] Using a difference-in-differences (DD) approach we thus compare the *difference* in changes in the consent rate for the intervention and control groups.[22–25] This method can be used to establish causal relationships in observational data as resulting estimates account for fixed differences between the treatment and control groups. Because we have longitudinal data, we can match respondents to themselves in the previous year by additionally including an individual-level fixed effect in the analysis, an indicator variable which accounts for all unobserved confounders which do not vary over time. The difference-in-differences approach involves implementing a linear probability model using the pooled individual-level datasets for the 2009 and 2010 HIV surveillance surveys.

The difference-in-differences approach we employ here has the advantage of allowing us to control for all fixed individual characteristics and implement a strategy for identifying the causal effect of being in the intervention group on consenting to participate in testing. The difference-in-differences approach makes the parallel trends assumption that the consent

trend over time for the intervention group would have been the same as that for the control group in the absence of the intervention. Given that the gift was allocated to families who were contacted in the last four weeks of surveillance in 2010 for operational reasons, rather than because of the characteristics of those families, the parallel trends assumption is very plausible in this case.

Summary statistics for variables used in the analysis are shown in **Table A1**. Full results from our preferred model specification are shown in **Table A2**. A summary of the DD estimates are shown in **Table 2** in the main text.

Robustness Checks

We carried out several tests to verify whether our results are robust. First, to confirm the linear probability model results in a binary choice model, we re-estimated models using logistic regression. We found an adjusted odds ratio of 5 (95% CI 5-7; p<0.001) for the main effect size, i.e., a highly significant five-fold increase in the odds of consenting to an HIV test due to the receipt of the voucher, supporting the linear probability model results. Second, we conducted the analysis at the individual level with an indicator as to whether the respondent received the voucher themselves (as opposed to their family), and found similar results (risk difference 29 percentage points, 95% CI 25-34 percentage points; p<0.001) for the combined sample of men and women. Finally, we re-ran the analysis on the 2010 data alone, finding results that were essentially the same as those generated by the main analysis (risk difference 25 percentage points, 95% CI 15-35 percentage points; p<0.001) for the combined sample of men and women. There were a small number of missing values on some covariates, and we included an indicator for missing values in the analysis. However, these variables did mostly not change over time and so were collinear with the individual fixed effects, and excluding them did not affect the results.

Web Extra Material Tables

Table A1 Sample descriptive statistics for 2010

	Median	Mean	SD	N
Consent to HIV Test	0	0.408	0.491	18,478
HIV Positive	0	0.239	0.427	7,462
Male	0	0.347	0.476	18,478
Age	38	40.997	18.924	18,478
In Food Voucher Intervention Group	0	0.181	0.385	18,478
Distance To Nearest Clinic (KM)	2.65	2.956	1.844	18,478
Distance To Nearest Secondary School	1.73	1.942	1.234	18,478
Distance To Nearest Primary School	1.13	1.204	0.692	18,478
Distance To Nearest Level 1 Road	4.85	6.931	6.556	18,478
Distance To Nearest Level 2 Road	1.13	1.439	1.2	18,478

**Type of Location**	No.	%
Peri-Urban	5,547	30
Rural	11,702	63.3
Urban	1,229	6.7
Total	18,478	100
**Month of Interview in 2010**		
January	861	4.7
February	2,181	11.8
March	2,676	14.5
April	2,054	11.1
May	2,174	11.8
June	710	3.8
July	780	4.2
August	1,923	10.4
September	1,637	8.9
October	1,641	8.9
November	1,464	7.9
December	377	2
Total	18,478	100
**Marital Status**		
Married	3,324	18
Polygamous	495	2.7
Divorced/Separated/Widowed	2,493	13.5
Engaged	326	1.8
Never Married	10,392	56.2
Under Legal Age	1,241	6.7
Missing/Other	207	1.1
Total	18,478	100

**Household has Electricity**	No.	%
Yes	11,788	63.8
No	3,477	18.8
N/A	2,917	15.8
Missing	296	1.6
Total	18,478	100
**Household Fuel Type**		
Electricity	9,025	48.8
Coal or Wood	4,725	25.6
Gas	880	4.8
Other	608	3.3
Missing	2,915	15.8
N/A	325	1.8
Total	18,478	100
**Household Asset Index**		
Quintile		
Lowest	2,998	16.2
2nd Lowest	3,006	16.3
Middle	3,216	17.4
2nd Highest	3,184	17.2
Highest	2,732	14.8
Missing	3,342	18.1
Total	18,478	100
**Education**		
None	2,530	13.7
Primary	2,083	11.3
Junior Secondary	2,362	12.8
Upper Secondary	6,649	36
Don't Know	1,592	8.6
Missing	3,262	17.7
Total	18,478	100
**Household has Running**		

**Mother is Alive**		
Dead	14,842	80.3
Alive	3,381	18.3
Missing	255	1.4
Total	18,478	100
**Father is Alive**		
Dead	14,853	80.4
Alive	3,291	17.8
Missing	334	1.8
Total	18,478	100
**Water**		
No	7,443	40.3
Yes	11,035	59.7
Total	18,478	100
**Household has Flush Toilet**		
No	17,245	93.3
Yes	1,233	6.7
Total	18,478	100

Note to table A1: Descriptive statistics for 2010 are shown. HIV prevalence is calculated on the basis of those consenting to test. All families in the last 10 weeks of the 40 week surveillance were allocated to receive an unconditional food gift voucher worth US$ 5 at the first contact with the family. The voucher was allocated in 2010 only.

Table A2 Full Regression Table for Consent to Test for HIV

Variables	All		Men		Women	
	Risk Difference (95%CI)	P-Value	Risk Difference (95%CI)	P-Value	Risk Difference (95%CI)	P-Value
Age	0.04 (-0.03 - 0.11)	0.22	-0.06 (-0.11 - -0.00)	0.03	0.01 (-0.07 - 0.09)	0.84
Age Squared	0.00 (0.00 - 0.00)	0.00	0.00 (-0.00 - 0.00)	0.07	0.00 (0.00 - 0.00)	0.01
**Urban (Omitted=Peri-urban)**						
Rural	0.04 (-0.12 - 0.21)	0.59	-0.09 (-0.31 - 0.14)	0.46	0.12 (-0.10 - 0.34)	0.27
Urban	0.03 (-0.16 - 0.22)	0.74	-0.04 (-0.43 - 0.36)	0.86	0.03 (-0.21 - 0.27)	0.80
Distance To Nearest Clinic	0.04 (-0.02 - 0.09)	0.19	0.10 (0.01 - 0.19)	0.03	0.01 (-0.06 - 0.08)	0.85
Distance To Nearest Secondary School	-0.00 (-0.08 - 0.08)	0.96	0.03 (-0.08 - 0.15)	0.57	0.02 (-0.08 - 0.12)	0.71
Distance To Nearest Primary School	0.03 (-0.07 - 0.13)	0.51	0.05 (-0.12 - 0.22)	0.54	0.02 (-0.10 - 0.14)	0.74
Distance To Nearest Level 1 Road	-0.02 (-0.04 - 0.00)	0.06	-0.02 (-0.04 - 0.01)	0.14	-0.03 (-0.05 - 0.00)	0.06
Distance To Nearest Level 2 Road	-0.03 (-0.09 - 0.04)	0.45	-0.24 (-0.35 - -0.14)	0.00	0.05 (-0.03 - 0.14)	0.21
**Marital Status (Omitted=Married)**						
Polygamous	-0.03 (-0.17 - 0.11)	0.69	0.05 (-0.12 - 0.22)	0.58	-0.06 (-0.24 - 0.12)	0.52
Divorced/Separated/Widowed	-0.00 (-0.10 - 0.10)	0.94	0.10 (-0.11 - 0.31)	0.34	-0.04 (-0.16 - 0.08)	0.50
Engaged	0.02 (-0.08 - 0.12)	0.74	0.05 (-0.10 - 0.19)	0.53	0.01 (-0.12 - 0.13)	0.93
Never Married	-0.02 (-0.09 - 0.06)	0.63	0.04 (-0.07 - 0.15)	0.43	-0.04 (-0.14 - 0.05)	0.38
Under Legal Age	0.00 (-0.08 - 0.08)	0.97	0.08 (-0.04 - 0.21)	0.17	-0.04 (-0.15 - 0.06)	0.43
Missing/Other	0.01 (-0.17 - 0.20)	0.88	0.12 (-0.13 - 0.37)	0.36	-0.03 (-0.29 - 0.22)	0.80
**Mother Alive (Omitted=Dead)**						
Alive	-0.00 (-0.05 - 0.05)	1.00	-0.03 (-0.12 - 0.07)	0.55	0.01 (-0.05 - 0.07)	0.74
Missing	0.00 (-0.14 - 0.14)	0.98	0.01 (-0.18 - 0.21)	0.89	-0.02 (-0.22 - 0.19)	0.88
**Father Alive (Omitted=Dead)**						
Alive	0.02 (-0.03 - 0.06)	0.47	0.05 (-0.03 - 0.12)	0.21	0.00 (-0.05 - 0.06)	0.92
Missing	-0.08 (-0.19 - 0.03)	0.16	-0.10 (-0.26 - 0.06)	0.22	-0.06 (-0.21 - 0.08)	0.40
**Electricty in House (Omitted=Yes)**						
No	-0.00 (-0.04 - 0.04)	0.96	0.02 (-0.05 - 0.08)	0.62	-0.01 (-0.06 - 0.04)	0.66
N/A	0.01 (-0.11 - 0.13)	0.86	0.04 (-0.17 - 0.25)	0.73	-0.01 (-0.15 - 0.14)	0.94
Missing	-0.03 (-0.28 - 0.22)	0.81	-0.03 (-0.38 - 0.32)	0.86	-0.02 (-0.42 - 0.38)	0.93
**Fuel in House (Omitted=Electric)**						
Coal/Wood	-0.00 (-0.04 - 0.03)	0.85	-0.00 (-0.06 - 0.05)	0.87	-0.00 (-0.04 - 0.04)	0.91
Gas	0.00 (-0.05 - 0.05)	0.99	-0.02 (-0.10 - 0.06)	0.60	0.02 (-0.04 - 0.07)	0.61
Other	0.01 (-0.05 - 0.07)	0.72	-0.03 (-0.13 - 0.07)	0.56	0.04 (-0.04 - 0.11)	0.34
Missing	-0.09 (-0.21 - 0.04)	0.18	-0.03 (-0.23 - 0.18)	0.81	-0.13 (-0.27 - 0.02)	0.10
N/A	-0.03 (-0.28 - 0.23)	0.82	0.04 (-0.31 - 0.38)	0.83	-0.09 (-0.50 - 0.32)	0.67
**HH Asset Quintile (Omitted=Lowest)**						
2nd	-0.01 (-0.05 - 0.03)	0.62	0.01 (-0.05 - 0.07)	0.78	-0.02 (-0.06 - 0.03)	0.50
3rd	-0.02 (-0.06 - 0.03)	0.54	0.00 (-0.08 - 0.08)	0.99	-0.02 (-0.08 - 0.04)	0.47
4th	-0.04 (-0.09 - 0.01)	0.15	-0.02 (-0.10 - 0.06)	0.64	-0.05 (-0.11 - 0.02)	0.16
5th	-0.03 (-0.09 - 0.02)	0.26	-0.02 (-0.10 - 0.07)	0.68	-0.04 (-0.11 - 0.03)	0.29
Missing	0.05 (-0.08 - 0.18)	0.44	0.00 (-0.20 - 0.21)	0.98	0.09 (-0.07 - 0.24)	0.29
**Education (Omitted=None)**						
Primary	0.01 (-0.03 - 0.06)	0.56	-0.04 (-0.12 - 0.04)	0.34	0.03 (-0.02 - 0.08)	0.19
Junior Secondary	0.00 (-0.04 - 0.05)	0.90	-0.02 (-0.10 - 0.06)	0.68	0.01 (-0.05 - 0.07)	0.76
Upper Secondary	0.02 (-0.03 - 0.06)	0.47	-0.01 (-0.08 - 0.07)	0.89	0.03 (-0.03 - 0.08)	0.30
Don't Know	0.01 (-0.03 - 0.05)	0.56	0.00 (-0.07 - 0.08)	0.97	0.02 (-0.03 - 0.07)	0.53
Missing	0.01 (-0.07 - 0.09)	0.79	-0.04 (-0.17 - 0.08)	0.50	0.04 (-0.05 - 0.14)	0.39
Running Water in House	0.02 (-0.02 - 0.05)	0.32	0.00 (-0.05 - 0.05)	0.95	0.02 (-0.01 - 0.06)	0.19
Inside Toilet	0.00 (-0.05 - 0.06)	0.94	0.01 (-0.07 - 0.09)	0.82	-0.00 (-0.07 - 0.06)	0.91
Year=2010	-0.08 (-0.16 - -0.01)	0.03			-0.03 (-0.12 - 0.07)	0.58

Year=2010*Intervention	0.25 (0.21 - 0.30)	0.00	0.27 (0.20 - 0.33)	0.00	0.25 (0.20 - 0.30)	0.00
Constant	-2.52 (-5.33 - 0.29)	0.08	1.74 (0.42 - 3.06)	0.01	-1.12 (-4.68 - 2.45)	0.54
Observations	36,956		12,836		24,120	
Number of Individuals	18,478		6,418		12,060	

Clustered Standard Errors in Parentheses

*** p<0.01, ** p<0.05, * p<0.1

Note to table A2: The model for table A2 is shown in equation A1. All households in the last 10 weeks of the 40 week surveillance were allocated to receive an unconditional food gift voucher worth US$ 5 at the first contact with the household. Consent data for individuals who were contacted to take a HIV test in 2009 and 2010 was pooled and the effectiveness of the voucher is the difference in difference estimate associated with being in the intervention group*2010. Standard errors are adjusted for clustering at the household level. The model includes a fixed effect for each individual, fixed effects for location, and fixed effects for month of interview which are not shown in the table. Characteristics which do not change over time are absorbed by the individual level fixed effect. Of the total 18,478 individuals in the analysis sample, 3,340 (18%) were in the intervention group. Of the total 6,418 men in the analysis sample, 1,206 (19%) were in the intervention group. Of the total 12,060 women in the analysis sample, 2,134 (18%) were in the intervention group.

Web Extra Material References

1. Hensen B, Taoka S, Lewis JJ, Weiss HA, Hargreaves J. Systematic review of strategies to increase men's HIV-testing in sub-Saharan Africa. *AIDS*. 2014;**28**(14):2133–2145.

2. Matovu JK, Makumbi FE. Expanding access to voluntary HIV counselling and testing in sub-Saharan Africa: alternative approaches for improving uptake, 2001–2007. *Trop Med Int Health*. 2007;**12**(11):1315–1322.

3. Mavedzenge SN, Baggaley R, Corbett EL. A review of self-testing for HIV: research and policy priorities in a new era of HIV prevention. *Clin Infect Dis*. 2013;**57**(1):126–138.

4. Pai NP, Sharma J, Shivkumar S, et al. Supervised and unsupervised self-testing for HIV in high- and low-risk populations: a systematic review. *PLoS Med*. 2013;**10**(4):e1001414.

5. Suthar AB, Ford N, Bachanas PJ, et al. Towards universal voluntary HIV testing and counselling: a systematic review and meta-analysis of community-based approaches. *PLoS Med*. 2013;**10**(8):e1001496.

6. Pettifor A, MacPhail C, Nguyen N, Rosenberg M. Can money prevent the spread of HIV? A review of cash payments for HIV prevention. *AIDS Behav*. 2012;**16**(7):1729–1738.

7. Lee R, Cui RR, Muessig KE, Thirumurthy H, Tucker JD. Incentivizing HIV/STI testing: a systematic review of the literature. *AIDS Behav*. 2014;**18**(5):905–912.

8. Thornton RL. The demand for, and impact of, learning HIV status. *Am Econ Rev*. 2008;**98**(5):1829.

9. Heyman J, Ariely D. Effort for payment a tale of two markets. *Psychol Sci*. 2004;**15**(11):787–793.

10. Brock JM, Lange A, Leonard KL. Giving and Promising Gifts: Experimental Evidence on Reciprocity from the Field. 2013;

11. Baird S, McIntosh C, Özler B. Cash or condition? Evidence from a cash transfer experiment. *Q J Econ*. 2011;**126**(4):1709–1753.

12. Deci EL. Effects of externally mediated rewards on intrinsic motivation. *J Pers Soc Psychol*. 1971;**18**(1):105.

13. De Walque D, Dow WH, Nathan R, et al. Incentivising safe sex: a randomised trial of conditional cash transfers for HIV and sexually transmitted infection prevention in rural Tanzania. *BMJ Open*. 2012;**2**(1).

14. Emanuel EJ, Currie XE, Herman A. Undue inducement in clinical research in developing countries: is it a worry? *The Lancet*. 2005;**366**(9482):336–340.

15. Kube S, Maréchal MA, Puppea C. The currency of reciprocity: Gift exchange in the workplace. *Am Econ Rev*. 2012;**102**(4):1644–1662.

16. Yan Y. The flow of gifts: Reciprocity and social networks in a Chinese village. Stanford University Press; 1996.

17. Berking H. Sociology of giving. Sage; 1999.

18. Fafchamps M. Solidarity networks in preindustrial societies: Rational peasants with a moral economy. *Econ Dev Cult Change*. 1992;147–174.

19. Posner RA. Theory of Primitive Society, with Special Reference to Law, A. *JL Econ*. 1980;**23**:1.

20. Marshall L. Sharing, talking, and giving: Relief of social tensions among! Kung Bushmen. *Africa*. 1961;**31**(03):231–249.

21. Tanser F, Hosegood V, Bärnighausen T, et al. Cohort Profile: Africa Centre Demographic Information System (ACDIS) and population-based HIV survey. *Int J Epidemiol*. 2008;**37**(5):956–962.

22. Farrar S, Yi D, Sutton M, Chalkley M, Sussex J, Scott A. Has payment by results affected the way that English hospitals provide care? Difference-in-differences analysis. *BMJ*. 2009;**339**:b3047.

23. Imbens GW, Wooldridge JM. Recent Developments in the Econometrics of Program Evaluation. *J Econ Lit*. 2009;**47**(1):5–86.

24. Athey S, Imbens GW. Identification and inference in nonlinear difference-in-differences models. *Econometrica*. 2006;**74**(2):431–497.

25. Branas CC, Cheney RA, MacDonald JM, Tam VW, Jackson TD, Ten Have TR. A difference-in-differences analysis of health, safety, and greening vacant urban space. *Am J Epidemiol*. 2011;**174**(11):1296–1306.

26. Tanser F, Bärnighausen T, Cooke GS, Newell M-L. Localized spatial clustering of HIV infections in a widely disseminated rural South African epidemic. *Int J Epidemiol*. 2009;**38**(4):1008–1016.
